# Clinical and cost-effectiveness of paramedic administered fascia iliaca compartment block for emergency hip fracture (RAPID 2)—protocol for an individually randomised parallel-group trial

**DOI:** 10.1186/s13063-022-06522-3

**Published:** 2022-08-17

**Authors:** Mark Kingston, Jenna Jones, Sarah Black, Bridie Evans, Simon Ford, Theresa Foster, Steve Goodacre, Marie-Louise Jones, Sian Jones, Leigh Keen, Mirella Longo, Ronan A. Lyons, Ian Pallister, Nigel Rees, Aloysius Niroshan Siriwardena, Alan Watkins, Julia Williams, Helen Wilson, Helen Snooks

**Affiliations:** 1 Swansea University, Swansea, UK; 2 South Western Ambulance Service NHS Foundation Trust, Exeter, UK; 3 Swansea Bay University Health Board, Port Talbot, UK; 4 East of England Ambulance Service NHS Trust, Melbourn, UK; 5 The University of Sheffield, Sheffield, UK; 6 Welsh Ambulance Services NHS Trust, St Asaph, UK; 7 Cardiff University, Cardiff, UK; 8 University of Lincoln, Lincoln, UK; 9 South East Coast Ambulance Services NHS Foundation Trust, Crawley, UK; 10 Royal Surrey County Hospital NHS Foundation Trust, Guildford, UK

**Keywords:** Hip fracture, Prehospital, Randomised controlled trial, Paramedic, Analgesia, Nerve block, Emergency medical services, Fascia iliaca compartment block

## Abstract

**Background:**

Approximately 75,000 people fracture a hip each year in the UK. This painful injury can be devastating—with a high associated mortality rate—and survivors likely to be more dependent and less mobile. Pain relief at the scene of injury is known to be inadequate. Intravenous morphine is usually given by paramedics, but opioids are less effective for dynamic pain and can cause serious side effects, including nausea, constipation, delirium and respiratory depression. These may delay surgery, require further treatment and worsen patient outcomes. We completed a feasibility study of paramedic-provided fascia iliaca compartment block (FICB), testing the intervention, trial methods and data collection. The study (RAPID) demonstrated that a full trial was feasible. In this subsequent study, we aim to test safety, clinical and cost-effectiveness of paramedic-provided FICB as pain relief to patients with suspected hip fracture in the prehospital environment.

**Methods:**

We will conduct a pragmatic multi-centre individually randomised parallel-group trial, with a 1:1 allocation between usual care (control) and FICB (intervention). Hospital clinicians in five sites (paired ambulance services and receiving hospitals) in England and Wales will train 220 paramedics to administer FICB. The primary outcome is change in pain score from pre-randomisation to arrival at the emergency department. One thousand four hundred patients are required to find a clinically important difference between trial arms in the primary outcome (standardised statistical effect ~ 0.2; 90% power, 5% significance). We will use NHS Digital (England) and the SAIL (Secure Anonymised Information Linkage) databank (Wales) to follow up patient outcomes using routine anonymised linked data in an efficient study design, and questionnaires to capture patient-reported outcomes at 1 and 4 months. Secondary outcomes include mortality, length of hospital stay, job cycle time, prehospital medications including morphine, presence of hip fracture, satisfaction, mobility, and NHS costs. We will assess safety by monitoring serious adverse events (SAEs).

**Discussion:**

The trial will help to determine whether paramedic administered FICB is a safe, clinically and cost-effective treatment for suspected hip fracture in the pre-hospital setting. Impact will be shown if and when clinical guidelines either recommend or reject the use of FICB in routine practice in this context.

**Trial registration:**

ISRCTN15831813. Registered on 22 September 2021.

## Administrative information

Note: the numbers in curly brackets in this protocol refer to SPIRIT checklist item numbers. The order of the items has been modified to group similar items (see http://www.equator-network.org/reporting-guidelines/spirit-2013-statement-defining-standard-protocol-items-for-clinical-trials/).

**Table:** 

Title {1}	Randomised trial of clinical and cost effectiveness of Administration of Prehospital fascia Iliaca compartment block for emergency hip fracture care Delivery (RAPID 2)
Trial registration {2a and 2b}.	Registered: 22/09/21 ISRCTN15831813 https://doi.org/10.1186/ISRCTN15831813.
Protocol version {3}	Version 1.2 25/03/2022
Funding {4}	Funded by the National Institute for Health Research Health Technology Assessment programme, project number 129972
Author details {5a}	^1^ Swansea University Medical School, ^2^ South West Ambulance Services NHS Foundation Trust ^3^ Swansea Bay University Health Board ^4^ East of England Ambulance Service NHS Trust ^5^ The University of Sheffield ^6^ Public and Patient Contributor ^7^ Welsh Ambulance Services NHS Trust ^8^ Cardiff University ^9^ University of Lincoln ^10^ South East Coast Ambulance Services NHS Foundation Trust ^11^ Royal Surrey County Hospital NHS Foundation Trust
Name and contact information for the trial sponsor {5b}	Research Engagement and Innovation Services, Swansea University, SA2 8PP—researchgovernance@swansea.ac.uk
Role of sponsor {5c}	This is an investigator-initiated trial. The Chief Investigator, within the sponsor organisation, is responsible for the trial design, collection, management, analysis and interpretation of data, writing of the report and publication decisions.

## Introduction

### Background and rationale {6a}

Hip fractures are very common—approximately 70,000 people suffer one each year in the UK [1]. A hip fracture can be devastating for a patient—there is a high associated short-term mortality, and those who survive are likely to be more dependent and less mobile than before their injury [2–4]. At present, a hip fracture is the most common cause of admission to an orthopaedic ward with an average length of stay of 14 days [1]. Patients with hip fracture occupy 2.5% of all hospital beds at any time and cost the National Health Service (NHS) £2 billion each year [5, 6]. As the average age of the population rises, the annual incidence and cost of hip fractures will also increase. Improving the care of patients with hip fracture is therefore of increasing importance.

When a patient fractures their hip, both the event itself and its aftermath are very painful [7]. Untreated pain will increase the neuro-hormonal stress response and the risk of delirium [8] but the literature suggests adequate pain relief is often not achieved for patients with hip fracture in the prehospital environment [9–11].

Intravenous (IV) opioids (usually morphine) are most commonly given to patients by paramedics at the scene of injury [6], but are relatively ineffective for dynamic pain (on movement), which patients commonly experience during transfer to hospital [5].

Importantly, opiates can cause numerous serious side effects, including nausea, constipation, delirium and respiratory depression. These may delay surgery, require further treatment and worsen patient outcomes [12]. Long-term outcomes of patients with hip fracture may improve if they did not receive opioids in prehospital care. If the paramedic who attends the patient is able to administer an alternative form of analgesia, the patient may not require morphine and thus not be exposed to opiate side effects [13–15]. For instance, if a patient who has received morphine experiences respiratory depression, they may require naloxone and be more likely to suffer from respiratory infections. Alternatively, if morphine causes the patient to be acutely confused, their surgery may be delayed beyond the recommended 48 h in which it is known to improve outcomes [16–18]. Such events lead to increased costs to the NHS, both directly from treatment required to alleviate the side effect, and from the increased length of hospital stay. Providing alternative, effective, non-opioid pain relief to patients with hip fracture in prehospital care may reduce side effects and improve patient outcomes including length of hospital stay (as found in an in-hospital study [19]). This would be beneficial for both patients and the NHS.

Fascia iliaca compartment block (FICB)—a local anaesthetic injection directly into the hip region—is routinely used by medical, and increasingly, nurse practitioners in the Emergency Department (ED) and on orthopaedic wards for pain relief. Although this procedure may provide effective analgesia as well as allow the reduction of morphine administration, it is not known whether it improves patient outcomes or is cost-effective in the prehospital setting.

FICB is a suitable alternative to opiate medication; it is a regional anaesthetic technique which delivers local anaesthetic directly to the hip region [20]. In-hospital studies have shown that FICB provides effective pain relief for hip fracture with minimal side effects (fewer than morphine) and is inexpensive to provide, and the technique is easy to learn [21–27]. The Association of Anaesthetists of Great Britain and Ireland support its delivery by non-medically trained health professionals [28]. So far, three small studies have been conducted, all demonstrating the viability of delivery of FICB by non-medics in prehospital care: one by nurses [29] and two by paramedics [30, 31]. One of these was a single-site feasibility study, RAPID, conducted by this study team, to ensure that this multi-centre randomised controlled trial (RCT) would be viable and worthwhile conducting [31].

## Aim and objectives {7}

Our aim is to test the safety, clinical and cost-effectiveness of paramedics providing FICB as pain relief to patients with suspected hip fracture in the prehospital environment.

## Trial design {8}

RAPID2 is a pragmatic multi-site, parallel group superiority randomised trial with an allocation ratio 1:1. RAPID2 is not a Clinical Trial of Investigational Medicinal Product (CTIMP) because the efficacy of local anaesthetics and indeed FICB has already been established. This was established with the MHRA for the purposes of the RAPID feasibility study.

## Methods: participants, interventions and outcomes

### Study setting {9}

The trial will be conducted in the prehospital environment in four ambulance service areas—three in England, one in Wales, and in ~ five receiving hospitals within these ambulance services’ catchments. The proposed ambulance service sites are East of England Ambulance Service NHS Trust, South East Coast Ambulance Services NHS Foundation Trust, South Western Ambulance Service NHS Foundation Trust and Welsh Ambulance Services NHS Trust. Data will be collected from both the ambulance services and receiving hospitals. There will be a Principal Investigator for each ambulance service and each receiving hospital.

### Eligibility criteria {10}

Our target population is patients with suspected hip fracture who are attended by emergency ambulance paramedics in response to a 999 call.

#### Inclusion for randomisation

Adult patients attended by a participating study paramedic following a 999 call who are:

- Assessed as having an isolated hip fracture—hip fracture assessment checklists will be provided to aid recognition, as in the feasibility study
- Conscious (Glasgow Coma Scale Score of ≥ 13)
- Haemodynamically stable
- To be conveyed to a participating receiving hospital

#### Exclusion prior to randomisation

Patients who.

- Are taking anticoagulants
- Have a hip prosthesis on the affected side
- Refuse analgesia
- Are thought to be having a stroke
- Are combative
- Are attended by a paramedic working alone

### Paramedic training

Paramedics will need to successfully complete training to administer the FICB. Letters of access will be arranged for paramedics to conduct training on hospital grounds. We will follow methods used in the RAPID feasibility trial, with additions prompted by qualitative work with paramedics in that study [32]. We therefore add ‘scenario training’ to group sessions, familiarisation with the trial packs and methods and refresher training midway through the recruitment period in order to prevent skill decay.

The three elements to the initial paramedic training are:

- 1) Online e-learning, covering administration of FICB and Good Clinical Practice training.
- 2) Group ‘classroom’ sessions, led by a consultant anaesthetist and covering hip fracture recognition, anatomy, pharmacology, FICB procedure and equipment, toxicity recognition and treatment, pre-hospital scenarios and trial processes. Life-sized groin models, custom made for training paramedics in the study, will be used to simulate administering FICB. A study site researcher will cover trial methods, including trial eligibility criteria, the importance of recording pain scores and the use of scratchcards and randomisation log. In the context of COVID, these training sessions may take place online.
- 3) In-hospital training. Pairs of paramedics will attend sessions at the receiving hospital where they will administer FICB to real patients, supervised by an anaesthetist or emergency medicine doctor. They will alternate between administering and critiquing the FICB to ensure their learning is active [33]. The paramedics must administer three FICBs competently and critique three FICBs performed by their peers, before being allowed to recruit patients to the study. The FICB and Intralipid (antidote for local anaesthetic toxicity) packs will be available for paramedics to familiarise themselves with. The anaesthetists providing training will run through prehospital scenarios with the paramedics, including assessing eligibility and taking consent. We will provide written material and assessments to optimise learning in between block performances. These will include research and clinical questions, e.g. patient trial eligibility, contraindications to FICB, information to give when taking consent for FICB including risks and how long the FICB will take to work.

We will make training documents available in an online area accessible to the training paramedics. Formal refresher training will be available to paramedics who have not performed FICB for more than 3 months.

Information sheets will be provided to all untrained operational staff (paramedics, advanced paramedic practitioners and emergency medical technicians) in the participating areas of ambulance services so that front line staff are aware of the trial and have an acceptable understanding of it.

### Who will take informed consent? {26a}

In this RCT in the emergency prehospital setting, we do not propose to attempt to consent patients to participate in research at the time of their injury, as they are likely to be in significant pain. We believe that truly informed consent to research cannot be gained in this highly emotional and distressing situation [34]. Paramedics will consent patients to treatment only, and an NHS researcher will approach the patient or consultee for consent to follow up through research questionnaires as soon as possible following the 999 call (and within 10 days).

### Additional consent provisions for collection and use of participant data and biological specimens {26b}

**Fig. 1:**
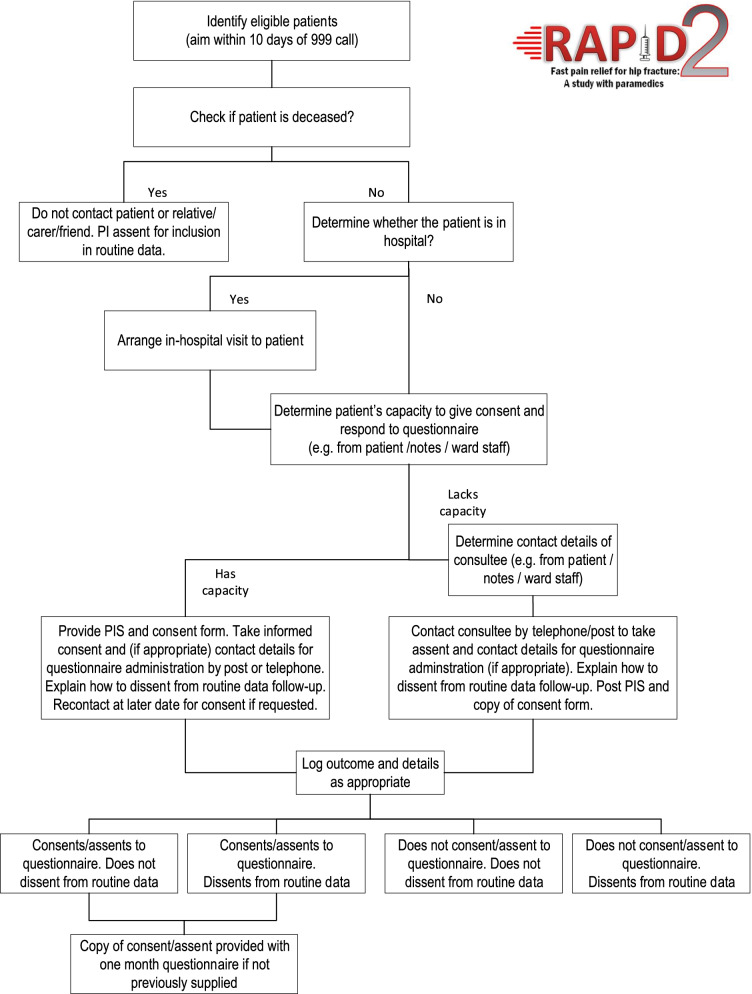
RAPID2 research consent flowchart

We expect a significant proportion of participants in RAPID2 to have cognitive impairment and lack capacity to give their own informed consent. We do not propose to exclude these patients from the trial, as the evidence we gather in this group may contribute to improving their care and outcomes. In these circumstances, we will seek consent from a consultee (which could be the patients’ relative, friend or carer). Our research consent model is outlined in Fig. 1.

### Interventions

#### Explanation for the choice of comparators {6b}

Research so far has suggested FICB is a suitable alternative to opiate medication for patients with hip fracture, but this has not been adequately tested in the prehospital environment.

#### Intervention description {11a}

##### Usual care

Currently, when a patient who has called 999 is attended by a paramedic for a suspected hip fracture, the paramedic clinically assesses the patient, takes their history, examines them and records observations (blood pressure, heart rate, respiratory rate, oxygen saturations, Glasgow Coma Scale, patient-reported pain score and temperature). Paramedics cannulate patients and provide IV fluids and/or oxygen, as appropriate, based on clinical parameters. They are currently able to provide systemic analgesia only, most commonly opioids (IV morphine), paracetamol and Entonox. In RAPID2, patients allocated to usual care will receive this care.

##### Intervention care

If the patient is randomly allocated to the intervention arm, the paramedic will administer FICB in addition to basic usual care as described above but avoiding use of opioids. The FICB will utilise 1% Prilocaine and will follow the method used in the RAPID feasibility study (based on Dalens et al. [20]). The paramedic will still provide the patient with paracetamol and Entonox but should not give the patient morphine for at least 20 min after the patient has received the FICB (to allow for the time of onset of Prilocaine). If, however, the FICB does not relieve the patient's pain after 20 min, the paramedic would be able to give the patient morphine if judged appropriate (‘rescue morphine’).

**Fig. 2:**
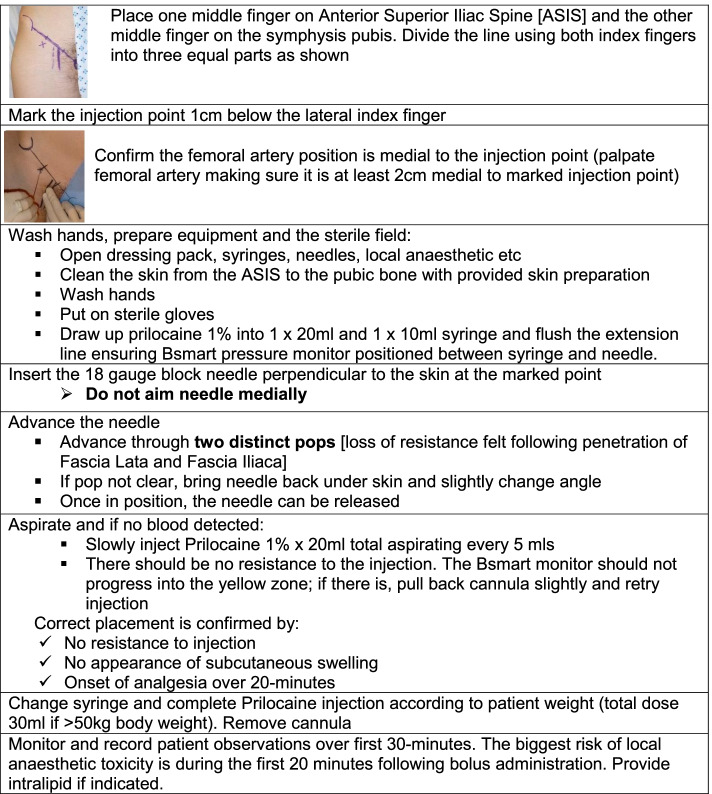
Treatment protocol for delivery of FICB (all materials/drugs supplied as part of RAPID2 Study Drugs Pack)

In order to provide FICB to a patient allocated to the intervention arm, the paramedic will:

- Assess for any contraindication to FICB
  - ○ Body mass apparently less than 50 kg
  - ○ Pregnancy
  - ○ Allergy to local anaesthetic
  - ○ Neurovascular damage to the affected leg
  - ○ Infection at the site of injection
  - ○ Previous femoral bypass surgery
  - ○ Inability to palpate the femoral artery on the affected leg
- Explain the risks and benefits of FICB
- Take verbal consent for the procedure
- Move the patient into a suitable position to administer the FICB
- Follow the treatment protocol for delivery of FICB (Fig. 2)

#### Criteria for discontinuing or modifying allocated interventions {11b}

*For hazard:* There are no pre-determined rules for stopping the trial due to hazard. However, the independent study Data Monitoring and Ethics Committee (DMEC) is expected to advise the Trial Steering Committee (TSC) if clear and consistent evidence emerges of a significant adverse effect or if, in the view of the DMEC, there is other compelling evidence of hazard that seems likely to outweigh any potential benefit.

*For benefit*: The DMEC will advise the TSC if, in its view, the study provides both (i) “proof beyond reasonable doubt” that the intervention improves the primary outcome and (ii) evidence that there are not likely to be material adverse effects on any other major morbidity. If, in the view of the DMEC, the evidence is not sufficiently convincing in one or more of the major subgroups, then it would not be expected to recommend stopping the trial early for efficacy.

#### Strategies to improve adherence to interventions {11c}

To support the change in practice clinically and operationally, we will address adherence through training, and site staff will monitor and promote intervention adherence through proactive and reactive measure. These will include regular communications with paramedics and trainers, provision of refresher training, auditing of suspected hip fracture cases, drug packs and scratch cards, and a prize draw entry for paramedics on randomising each eligible patient.

#### Relevant concomitant care permitted or prohibited during the trial {11d}

All participants can receive paracetamol and Entonox for pain relief. Our protocol states that patients randomly allocated to receive FICB can be provided with morphine only if the FICB has not provided adequate pain relief after twenty minutes.

#### Provisions for post-trial care {30}

Care is provided within the UK National Health Service. Any compensation claims arising from the study would be dealt with by sponsor (Swansea University) public liability insurance, or NHS Indemnity Schemes as appropriate. In line with good practice, the study team will make aggregated results available to all participants [35]. These will be provided online, with access details in patient information documents.

### Outcomes {12}

We will compare outcomes between trial arms:

*Primary outcome*: change in patient-reported acute pain from initial paramedic assessment (pre-randomisation) to triage nurse assessment on arrival at ED.

*Secondary outcomes* during initial care and up to 4 months:

•Routine data

- Ambulance service job cycle time (from 999 call to ‘ambulance free’)
- Analgesia and anti-emetics administered prehospitally, including morphine and ‘rescue morphine’
- Length of stay in hospital, ITU and residential rehabilitation care following injury
- Subsequent ED attendances and emergency admissions
- Mortality
- Diagnosis (for patients who did not have a hip fracture)
- Where patient was admitted from and discharged to

•Patient-reported outcomes

- Satisfaction with care (Quality of Care Monitor at 1 month)
- Health-related quality of life (HRQoL) (EQ-5D-5L at 1 and 4 months)
- Mobility (Rivermead Mobility Index at 1 and 4 months. One question will be removed to enable the patient to complete the questionnaire by themselves)

•Costs to the NHS

### Participant timeline {13}

Day 0—Participant randomly allocated to trial arm and receives experimental or usual care.

Day 0–day 7—Participant will be monitored for SAEs.

By day 10—A trained NHS researcher will make contact with the participant to explain that they have been recruited to a research trial, seek the participant’s consent for questionnaire follow-up, offer the chance to dissent from anonymised routine follow-up and to answer any questions the participant may have.

Day 28—Participant receives a study questionnaire which includes:

- Quality of Care Monitor
- EQ-5D-5L
- Modified Rivermead Mobility Index

Day 120—Participant receives a study questionnaire which includes:

- EQ-5D-5L
- Modified Rivermead Mobility Index
- £10 High Street Shopping Voucher

### Sample size {14}

The RAPID feasibility study used an 11-point pain scale (0 being no pain, 10 the worst pain imaginable), and reported an average reduction of approximately 4 points in pain score (standard deviation 2.7 points) [31]. These data consistent with that reported elsewhere in broadly similar settings [30]. Our patient and public contributors and clinicians judged an average difference in change of 0.5 ~ 0.6 points to be clinically important. The mid-point of this range in average differences in change corresponds to a standardised statistical effect of ~ 0.2 between control and intervention arms; for 90% power at the 5% significance level. We therefore need approximately 1000 analysable outcomes. If approximately 20% of patients lack pain scores, 10% of participants dissent from anonymised follow-up of routine records, and we are unable to match 1% of cases in Secure Anonymised Information Linkage (SAIL) and NHS Digital, then we will need to allocate randomly and equally 1404 patients to study arms.

The rate of recruitment of 5 patients randomised per paramedic per year observed in our feasibility study will be reduced by the addition of an exclusion criterion (the use of anticoagulants). Our data indicate that the reduction is likely to be ~ 30%. With this reduction, we have calculated that we will need five hospital sites with approximately 40 trained paramedics recruiting an average of seven patients in 24 months. Each site will be expected to recruit approximately 280 patients in this time, although there may be slightly different recruitment rates between sites due to the different demographics and sizes of catchment areas of receiving hospitals.

### Recruitment {15}

To support paramedic recruitment we will advertise the trial in each participating ambulance service using communication methods tailored to each site (e.g. email, Twitter, the intranet and posters). Paramedics will be advised to contact a local (research funded) site researcher to sign up for training.

We will monitor participant recruitment closely in the first 6 months of the trial to see if these targets are realistic, so that we can take action to rectify any problems identified. Although we expect that 40 paramedics are required in each site to meet our participant recruitment target, we will train 10% more at each site to account for attrition (due to maternity or sick leave, secondment or career change or progression).

**Fig. 3:**
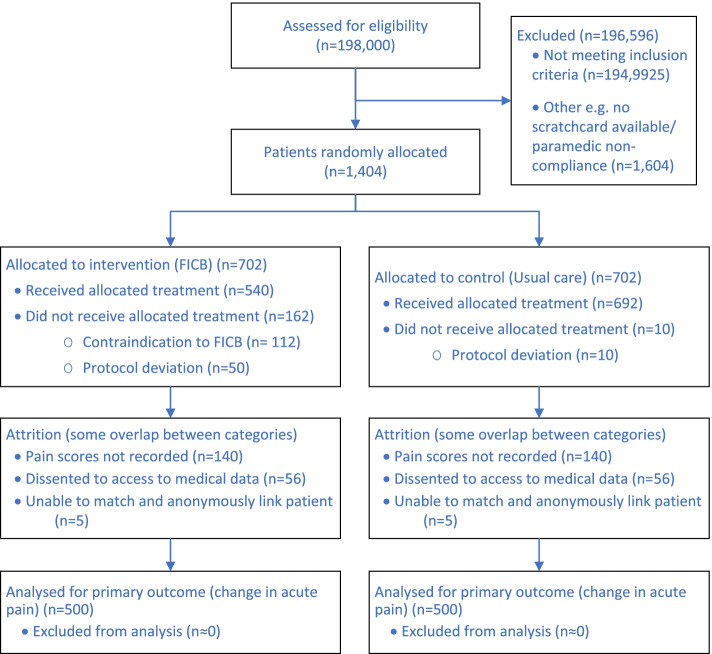
CONSORT flow of participants

Our projected participant recruitment is summarised in Fig. 3.

## Assignment of interventions: allocation

### Sequence generation {16a}

An independent statistician will produce a randomisation schedule, stratified by site and paramedic, with allocations (control/intervention) concealed on scratchcards.

### Concealment mechanism {16b}

Allocations will be concealed by using scratchcards. With a maximum of 44 paramedics per site, we will produce 2400 scratchcards and issue these in packs of ten. For eligible patients, paramedics will scratch the card’s panel to reveal ‘Intervention—FICB’ or ‘Control—usual care’ out of the sight of the patient. As we will use the scratchcard serial number as the basis of the patient’s study ID, the paramedic will retain the scratchcard in order to store it with a randomisation log at their ambulance station, so that the site researchers can monitor recruitment. Site researchers will conduct an audit of scratchcards at intervals during the recruitment period, and again at the close of recruitment.

### Implementation {16c}

The scratchcards will be produced by staff at Swansea University and sent securely to the site researchers within each ambulance service site.

## Assignment of interventions: blinding

### Who will be blinded {17a}

This is an open trial, as it would not be possible to blind paramedics or patients to the treatment they received as sham FICB would be unethical [36]. To reduce the risk of bias in reporting pain scores, clinical staff will be blinded to the patient’s allocation when recording pain scores:

- The paramedics will be instructed to record the patient’s baseline pain score before randomisation
- The triage nurse in the ED will be instructed to take the second pain score at handover, before the paramedic reveals which arm of the trial the patient was allocated to.

### Procedure for unblinding if needed {17b}

#### Periodic review of unblinded data

During the study, summaries of all serious adverse events (SAEs) and other study outcomes will be supplied in strict confidence the DMEC. These will be circulated to DMEC Members at least a week in advance of each meeting. The DMEC Chair will be able to request additional analyses (i.e. analyses not contained in the usual report) subject to agreement from the TSC Chair.

## Data collection and management

### Plans for assessment and collection of outcomes {18a}

We will use routinely gathered data wherever possible.

1. *Data related to index event and episode of care:* Site researchers will collect prehospital data for all patients from their patient clinical record (PCR). This will include pre-randomisation patient-reported pain score; job cycle time (from first 999 call for the incident to time ambulance reported free to respond to next 999 call); medications given (i.e., anti-emetics and analgesia—FICB, paracetamol, Entonox and morphine); and any immediate complications of analgesia given. The researcher will collect data from the ED, most importantly pain score on arrival there. The researcher will check local incident reporting mechanisms (for example, Datix) for any serious adverse events. We are particularly interested in adverse events which may be due to the FICB being performed in the prehospital environment, for example, an increased incidence of infection at the injection site. The researcher will also collect information about any complications of the FICB from the medical notes and how long the patient waited to be taken to theatre for surgical fixation from the hospital’s theatre system. We will record data regarding the patient’s diagnosis, so that we know what injury the patient did have, if not a hip fracture. Each site researcher will be given training and guidance on completion of the Case Report Forms (CRF). We will monitor completion rates and report back to local teams. All data collection, handling and storage will be compliant with data protection policies, including UK General Data Protection Regulations (GDPR) [37]. We will train, monitor and support paramedics and triage nurses in ED to complete pain scores as reported by patients. We have included costs to ensure that training and support is offered at the outset and throughout the trial at all study sites.
2. *Patient reported outcome measures:* We will send questionnaires to patients at 1 and 4 months by post (unless they are still in hospital, in which case they can be completed face-to-face). Patients (or and identified consultee) will be telephoned approximately 3 days after they have been sent the questionnaire to ask if they would prefer to answer the questionnaire over the telephone or send back the questionnaire. If we do not receive the questionnaire from the patient three weeks after sending it, we will send a reminder letter to the patient. Questionnaire responses will allow us to compare patient satisfaction (Quality of Care Monitor) [38], HRQoL (EQ-5D-5L) [39] and mobility (Rivermead Mobility Index [40]) between patients in each arm of the trial. Before contacting patients, we will check records to ensure that the patient has not died to avoid causing distress to their families, as well as to record patient mortality. There will be a space on the questionnaire to indicate whether it has been completed by the patient or by a consultee, so that outcome data can still be collected for patients with cognitive impairment.
3. *Anonymised linked outcomes:* We will link CRF and patient-reported outcome data to nationally held routine data through NHS Digital (in England) and Digital Health and Care Wales (in Wales) using the split file technique [41] so that no identifiable data are held by the central Swansea Trials Unit team. Trial data will be stored and securely available for analysis in the SAIL Gateway. We have successfully used this approach several times before, e.g. SAFER 2 and PRISMATIC [42, 43].

We will request individual-level data on previous hip fractures (up to 5 years before recruitment) from records held within SAIL/NHS Digital and use these to define appropriate baseline covariates for statistical models when making adjusted comparisons between trial arms. Subject to appropriate ethical, research and information governance permissions, we will also request data on secondary outcomes related to diagnoses; disposition from ED; length of stay at index episode in hospital, ITU and residential rehabilitation ward; further ED attendances and emergency admissions; and total length of stay and deaths up to 4 months.

Our outcomes and measurement intervals match those used in the National Hip Fracture Database (NHFD) as far as possible, but it is important to note that our population will be different, as approximately 20% of our participants may not have a hip fracture.

### Monitoring for false positives

The positive predictive value of the paramedics’ diagnoses of hip fracture in the feasibility study was slightly lower (80.7%) than desirable in practice [44]. Therefore, we will monitor for false positives and discuss these with paramedics on a regular basis to ensure that they are aware of incorrect diagnoses and are able to learn from them.

### Health economics

The health economics strand embedded within RAPID2 includes three interlinked aims: (a) to cost the intervention, (b) to measure patient’s NHS resource use from baseline to end of follow-up, and (c) to determine the value for money of the new model of care via cost-effectiveness analysis (CEA), cost utility analysis (CUA) and cost and consequences analysis (CCA) [45].

#### Intervention costs

This includes all the costs (excluding research costs) sustained to deliver the intervention. A purposely designed data collection questionnaire tested in the feasibility study will be sent to each recruiting site to retrieve information about time spent in training, travel costs and equipment—including groin models and FICB packs. Compared to the feasibility study, the detailed costing of the intervention will benefit from the multi-centre nature of the study and give a more representative picture of NHS costs for wider implementation.

#### NHS resource use

Data sources on NHS resource use in follow-up comprise the CRF validated in the RAPID feasibility study and routine data. From these, we will retrieve the following information: hospital stays, hospital-based treatments, readmissions, ED attendances, AEs (e.g. deep vein thrombosis), ARs (e.g. infection), SAEs (e.g. pneumonia), SUSARs (e.g. femoral nerve damage), prescription of non-opioid analgesia (including FICB administered by paramedics), prescription of opioids and prescription of anti-emetic. Resource use will be costed using appropriate unit cost data (Unit costs of health and social care, Personal Social Services Research Unit, NHS reference costs, Department of Health, British National Formulary).

#### Outcome measures for cost-effectiveness analysis (CEA) and cost utility analysis (CUA)

The data collection process of Pain scores and EQ-5D-5L is reported in the subsection on routine data collection.

### Plans to promote participant retention and complete follow-up {18b}

Patients and consultees will be sent a £10 High Street Shopping voucher with the 4-month questionnaire as an incentive, which has been shown to increase response rates [46, 47].

### Data management {19}

Data on CRFs will be entered onto a REDCap trial database at each site. There will be range checks put in place on REDCap for certain data values to randomise errors, e.g. only dates within the recruitment period can be used for date of randomised on. Quality assurance checks will be carried out on 10% of the data inputted at each site. If any errors are found, that site’s data entry will be checked in full. At the end of the recruitment period, identifiable and clinical data in split file format will be exported to NHS Digital (England) or Digital Health and Care Wales by research support staff to be randomised and then exported in to the SAIL (Secure Anonymised Information Linkage) databank for secure storage and analysis via the SAIL gateway [41]. Irreversibly randomised data such as these are not considered personal data under the General Data Protection Regulation (GDPR; EU Regulation 2016/679). However, until the data are exported and randomised, we will include instructions on how to dissent from the study prior to data export when the NHS researcher approaches the patient to discuss the trial up to 10 days after their injury. This will mean that participants will have until the end of the recruitment period to contact research support staff to request their data not be made available to the study team. We will store SAE data separately from the rest of the trial data, as we will report safety for all randomised patients.

### Confidentiality {27}

Personal information will be required to monitor for SAEs and to send the participants questionnaires. All data will be stored in accordance with Good Clinical Practice. No identifying images or other personal or clinical details of participants are presented here or will be presented in reports of the trial results.

## Statistical methods

### Statistical methods for primary and secondary outcomes {20a}

#### Data analysis

The primary analysis will be by ‘treatment allocated’, adjusting for explanatory factors and covariates including patient age and gender, patterns of presentation (e.g., whether out-of-hours or not) and previous hip fractures. Generalised multilevel mixed linear models will accommodate clustering effects for paramedics and study sites, with numbers of levels in models determined using statistically significant changes in likelihood ratio tests according to the principle of parsimonious parameterisation. Residual diagnostics will be used where analyses assume normality; if the distributions of residuals are markedly non-normal (e.g. marked skewness in the primary pain outcome), data transformation techniques or bootstrapping will be considered. Residual analysis will also be used to identify outliers; identified outliers will be excluded before repeating the analysis.

#### Health economic data analysis

The health economic analysis, also by treatment allocated, will be carried out in line with the NICE guidelines on health technology appraisal and presented in accordance to the CHEERS checklist [48]. Management of missing and non-normally distributed economic data will follow the principles outlined above.

##### *Analysis of training costs*

An average cost per person trained will then be determined by dividing the total cost of the training by the number of attendees. The cost per patient treated will be determined by dividing the training cost by the likely number of eligible patients seen by each ambulance unit trained. Training is a capital investment with a 5-year life expectancy and, as such, the cost will be annuitised (using 3.5% discount rate) to determine the cost per year.

##### *Cost-effectiveness analysis and cost utility analysis*

We will conduct two main analyses of incremental cost-effectiveness to estimate the incremental cost per unit change in pain score from that recorded pre-randomisation to that recorded on arrival in ED. Compared to hospital setting (both ED and ward), pain is relatively rarely used as a primary outcome measure in the pre-hospital setting. This large multi-centre study will offer some useful insights into its use as an outcome measure in this setting. We again use mixed linear models to estimate the cost-effectiveness ratios and employ non-parametric bootstrap estimates (bias corrected) to confirm 95% confidence intervals. A cost-effectiveness plane will present the probability that the intervention is dominant or cost-effective. If FICB is more effective but also more expensive, the cost-effectiveness acceptability curves (CEAC) will show the probability of effectiveness against different thresholds of willingness to pay for pain reduction [49].

The incremental cost utility analysis will assess the cost per quality-adjusted life years (QALYs) of the new model of care. In the UK, the National Institute for Health and Care Excellence (NICE) recommends the use of quality-adjusted life years (QALYs) as a measure of health benefits and the use of the generic preference-based HRQoL measure EQ-5D-5L to determine health status. HRQoL appears to be sensitive to change and appropriate for use in orthopaedic patients [50–53]. Again, mixed linear models will be used to estimate the cost utility ratios and bias-corrected non-parametric bootstrap is used to confirm 95% confidence intervals around the point estimate. We will use baseline EQ-5D-5L scores as a covariate in the estimation of QALYs.

A series of sensitivity analyses, involving NHS cost drivers and training formats, will assess the robustness of both incremental cost-effectiveness analyses.

##### *Cost and consequences analysis*

In this analysis costs are set against the whole range of outcomes (primary and secondary). This framework of analysis is now recognised as a useful alternative by NICE when carrying out economic evaluation with multiple important outcomes, interventions that have multiple effects which are difficult to summarise in a common unit such as public health intervention (NICE 2013) and the preferred framework for the economic evaluation of public health interventions. Because cost and consequences analyses are not restricted to a single outcome measure, the use of this framework will enable us to focus the attention of policy makers to the set of secondary outcomes we are measuring in this study [54].

We will formalise and agree with trial management and oversight committees in advance of any analysis, all planned analyses in a combined Statistics and Health Economics Analysis Plan (SHEAP), compliant with relevant Swansea Trials Unit Standard Operating Procedures. The SHEAP will provide full details on model fitting conventions, such as inclusion and exclusion rules for covariates and factors, management of missing data, and the reporting of outcomes. In summary: potential factors and covariates to be included in models will be tested; those with an F value of less than 1 (that is, they increase the standard error of the estimate) will be excluded and the analysis recalculated. Binary covariates where almost all cases (> 90%) are in one category will also be excluded. Wherever possible, outcome descriptions, summaries and comparisons will be reported using appropriate CONSORT guidelines, including estimates with 95% confidence intervals (allowing two-tailed tests at the 5% significance level).

#### Interim analyses {21b}

No interim analyses are planned.

### Methods for additional analyses (e.g. subgroup analyses) {20b}

#### SPIRIT guidance: methods for any additional analyses (e.g. subgroup and adjusted analyses)

A subgroup analysis may compare outcomes by study arm for patients with a hospital diagnosis of hip fracture vs those patients with any other diagnosis.

### Methods in analysis to handle protocol non-adherence and any statistical methods to handle missing data {20c}

#### SPIRIT guidance: definition of analysis population relating to protocol non-adherence (e.g. as randomised analysis), and any statistical methods to handle missing data (e.g. multiple imputation)

Missing data will be handled using multiple imputation. However, where data is missing because a patient opted out of, e.g. follow-up using routine data, that patient will instead be excluded from those outcome measures.

Multiple imputation will be performed as a single imputation run including the following variables: age, sex, ethnicity, deprivation score, pain score prior to randomisation, pain score at ED, pre-hospital analgesia/anti-emetics, mortality, diagnosis, admission location, discharge location, subsequent ED attendances, subsequent emergency admissions, satisfaction with care, health-related quality of life, mobility, ambulance job cycle time, length of hospital stay, length of ITU stay and length of residential rehabilitation.

### Plans to give access to the full protocol, participant-level data and statistical code {31c}

The full protocol, participant-level data and statistical code are available upon reasonable request.

## Oversight and monitoring

### Composition of the coordinating centre and trial steering committee {5d}

The trial is coordinated by Swansea University’s Health Services Research team. This includes a core team meeting regularly (CI, trial manager, statistician, data manager, administrator). A full trial management group (TMG) includes the core team and all co-applicants and meets at least every 3 months. A separate sub-group coordinates data management (core team plus health economics lead).

The RAPID2 TSC is a multidisciplinary group providing independent expertise and scrutiny and comprising the following members who meet every 6 months.

- An independent Chair (and statistician)
- Four independent clinician(s) or Scientist(s) with relevant experience (paramedic, Consultant of Emergency Medicine, Anaesthetist, Health Economist)
- Two Public Contributors.

### Composition of the data monitoring committee, its role and reporting structure {21a}

The independent Data Monitoring and Ethics Committee (DMEC) also meets every 6 months and reports to the TSC. It comprises:

- Health Economist (Chair), Statistician, Anaesthetist, Paramedic, Nurse.

The DMEC will monitor study data at interim periods and make recommendations to the Trial Steering Committee (TSC) on whether there are any ethical or safety reasons why the trial should not continue. Its members will have access to comparative data and may request the un-blinding of such data at any time. The DMEC will also consider requests for the release of data. The DMEC may be asked by the TSC, Trial Sponsor or Study Funder to consider data emerging from other related studies. If new evidence becomes available during the course of the trial, it is the responsibility of the trial and/or Data Manager to provide that information to the DMEC to allow them to consider such issues and make recommendations on the continuation of the trial to the TSC.

#### Public involvement

People affected by hip fracture, as patients or carers, are members of the TMG overseeing trial implementation (SJ, M-LJ). One was a co-applicant on the funding proposal. Along with members of a public involvement group [55], they were directly involved throughout development of the research design, in particular in selection of patient outcomes. We have recruited two additional individuals to join the independent Study Steering Committee of clinical, policy, academic, methodological and public contributor experts. We provide honoraria, briefings and other support as needed in line with best practice and report public involvement in our outputs [56, 57]. We have a named lead for public involvement in the team (BAE) who brings expertise and experience to this role.

### Adverse event reporting and harms {22}

We will monitor adverse events in all randomised patients up to 1 week, to assess them for seriousness and to investigate all serious adverse events (SAEs) to establish whether they are a reaction to the treatment received. We will report all suspected unexpected serious adverse reactions (SUSARs) promptly to the sponsor and Chair of the DMEC. We will report SAEs regularly to the Trial Management Group (TMG), TSC, DMEC and Sponsor.

### Frequency and plans for auditing trial conduct {23}

Site monitoring visits will be conducted at all sites at least four times during the recruitment period (approximately once every 6 months) by a member of the Swansea-based core team. Scratchcards will be audited at least four times during the recruitment period (approximately once every 6 months). This will be to ensure that they are being used in numerical order and the silver panels are not being tampered with.

### Plans for communicating important protocol amendments to relevant parties (e.g. trial participants, ethical committees) {25}

Changes to the protocol require approval from the funder, sponsor, Research Ethics Committee and NHS R&D offices. Any changes will be communicated widely through appropriate channels and site liaison.

## Dissemination plans {31a}

We have developed a communications, publications and dissemination plan including the assessment of stakeholder needs and communications activities and milestones. The plan includes engagement with patient and professional groups, NHS managers, commissioners and policy makers, including regular study newsletters We will produce lay summaries with our patient contributors where appropriate. We will disseminate findings through a website, journal articles, conferences and policy and stakeholder events.

## Discussion

The training planned in this RCT has been affected by Covid-19 in a number of ways. Early in the trial, before recruitment began, one ambulance service withdrew, as they felt their paramedics were fatigued from Covid-19 and did not want to give them additional work. Training has been delayed by Covid-19 for two main reasons: lack of elective hip replacement lists and reduced access to hospitals. Finally, Covid-19 has meant that we needed to modify the scratchcard design slightly so that they could be used wearing full personal protective equipment (PPE)—we are now including a ‘scratcher’ with the scratchcards.

In addition to Covid-19 delays, some hospitals have moved away from using landmark guidance to perform FICB, in preference of ultrasound. This is not something that can be done in the prehospital environment, so we could not conduct training in these hospitals.

## Trial status

Current protocol number 1.2. Participant recruitment is planned to commence from September and last 24 months.

## Data Availability

Any requests for the trial dataset following the trial will be considered by our statistician, Alan Watkins, in line with Swansea Trials Unit standard operating procedures for data privacy/sharing.

## Abbreviations

AE: Adverse events
ASIS: Anterior superior iliac spine
CCA: Cost and consequences analysis
CEA: Cost-effectiveness analysis
CEAC: Cost-effectiveness acceptability curves
CRF: Case Report Form
CTIMP: Clinical Trial of Investigational Medicinal Product
CUA: Cost utility analysis
DMEC: Data Monitoring and Ethics Committee
ED: Emergency department
FICB: Fascia iliaca compartment block
GDPR: General Data Protection Regulation
HRQoL: Health-related quality of life
IV: Intravenous
KG: Kilogram
MHRA: Medicines and Healthcare Products Regulatory Agency
ML: Millilitre
NHS: National Health Service
NICE: National Institute of Clinical Excellence
NIHR: National Institute for Health Research
PCR: Patient clinical record
QALY: Quality-adjusted life year
RCT: Randomised controlled trial
SAE: Serious adverse event
SAIL: Secure Anonymised Information Linkage
SHEAP: Statistical and Health Economics Analysis Plan
TMG: Trial Management Group
TSC: Trial Steering Committee
